# Direct contact between iPSC-derived macrophages and hepatocytes drives reciprocal acquisition of Kupffer cell identity and hepatocyte maturation

**DOI:** 10.7554/eLife.108938

**Published:** 2026-06-29

**Authors:** Christopher Zhe Wei Lee, Farah Tasnim, Xiaozhong Huang, Raman Sethi, Yoohyun Song, Tatsuya Kozaki, Sebastiaan De Schepper, Nicholas Ang, Ivy Low, You Yi Hwang, Jinmiao Chen, Hanry Yu, Florent Ginhoux

**Affiliations:** 1 Innovations in Food and Chemical Safety Programme (IFCS), A*STAR Singapore Singapore; 2 Singapore Immunology Network Singapore Singapore; 3 School of Biological Sciences, Nanyang Technological University Singapore Singapore; 4 Institute of Bioengineering and Bioimaging Singapore Singapore; 5 Department of Physiology, Yong Loo Lin School of Medicine, National University of Singapore Singapore Singapore; 6 Bioinformatics Institute (BII), Agency for Science, Technology and Research (A*STAR) Singapore Singapore; 7 Institute of Molecular and Cell Biology (IMCB), Agency for Science, Technology and Research (A*STAR) Singapore Singapore; 8 NUS Graduate School – Integrative Sciences and Engineering Programme (ISEP) Singapore Singapore; 9 Mechanobiology Institute Singapore Singapore; 10 CAMP IRG, Singapore-MIT Alliance for Research and Technology Singapore Singapore; 11 Shanghai Institute of Immunology, Shanghai JiaoTong University School of Medicine Shanghai China; 12 Translational Immunology Institute, SingHealth Duke-NUS Academic Medical Centre Singapore Singapore; 13 U1356 Next Generation Immuno-Oncology research, Université Paris-Saclay, Gustave Roussy Villejuif France; The Hebrew University of Jerusalem Israel; National Institute of Immunology India

**Keywords:** macrophage, hepatocyte, IPSC, Human

## Abstract

As the resident tissue macrophage of the liver, Kupffer cells (KCs) play an important role in homeostasis and tissue support. However, current in vitro liver models often ignore the contribution of these KCs towards the proper response and function of the tissue. This is especially relevant when we consider the implications of immune-mediated drug injuries. To address this issue, we developed an isogenic co-culture system utilising iPSC-derived macrophages (iMacs) and hepatocytes (iHeps). Directly co-culturing iHeps with iMacs improved the differentiation and maturation of the iHeps, with significant downregulation of fetal hepatocyte markers as well as upregulation of cytochrome genes. Furthermore, the co-culture also imparted stronger KC identity to the iMacs in a contact-dependent manner, with iMacs cultured in iHep conditioned media alone showing weaker expression of key KC markers. Finally, challenging the iHep-iMac co-culture system with seven paradigm hepatotoxic compounds showed dose-dependent cytokine response in the five compounds associated with immune-mediated liver injuries while no significant changes were observed in the two compounds with no reported immune-dependent complications. This effect was also not recapitulated when the co-culture was instead performed with human peripheral blood monocyte-derived macrophages, suggesting that iMacs are essential for liver toxicity response. Taken together, our study shows not only the importance of macrophages in tissue systems, but also that the source of macrophages is critical to the development of accurate in vitro human models.

## Introduction

Hepatic macrophages play key roles in immune-mediated hepatotoxicity, liver injury, and repair (Shan and Ju, 2020; Dixon et al., 2013). They are impacted by autocrine and paracrine signals from hepatic cells, especially hepatocytes. They also release soluble stress signals in response to external stimuli from foreign particles, leading to macrophage activation and production of cell signalling and stress pathway modulators, including reactive oxygen species and cytokines (Krenkel and Tacke, 2017; Blériot and Ginhoux, 2019). In return, hepatic macrophage activation also regulates parenchymal and non-parenchymal cell death and the metabolic activity of hepatocytes (Wen et al., 2021). Such crosstalk between liver parenchymal cells (hepatocytes) and non-parenchymal modulators (hepatic macrophages) is critical in modelling liver injury, especially immune-mediated drug-induced responses (Tasnim et al., 2021).

Two major populations of hepatic macrophages have been reported: liver-resident Kupffer cells (KCs) and monocyte-derived macrophages (MoMϕs) that infiltrate the liver upon insult (Krenkel and Tacke, 2017). While MoMϕs have been well studied in vivo and applied in in vitro models (Tasnim et al., 2021), in more recent years it has been revealed that KCs are derived from embryonic macrophages that seed the liver early on during development (Mass et al., 2016; Lee and Ginhoux, 2022), and are only minimally replaced by circulating monocytes under steady state conditions (Scott et al., 2016). This also implies that human peripheral blood monocytes, the conventional source of primary human macrophages, might not be able to fully recapitulate KC biology. However, it has been demonstrated that murine iPSC-derived macrophages (iMacs) more closely resembled embryonic macrophages than monocyte-derived macrophages (Takata et al., 2017), suggesting that human iMacs might also serve as a suitable analogue for human embryonic macrophages. Based on these new insights, in our previous work, we differentiated iMacs into iPSC-derived KCs (iKCs) using primary hepatocyte conditioned media (PHCM) to drive iMacs towards iKC identity (Tasnim et al., 2019). Although these iKCs partially resembled primary human KCs (PhKCs) based on the expression of KC-specific markers and drug response, PHCM is not readily available and might exhibit differences depending on donor variability of primary hepatocytes. Most importantly, the direct cellular interactions between iMacs and hepatocytes and their potential to impart better reciprocal identity and functionality to each other has not been studied before.

We recently generated macrophage-sufficient brain organoids by coculturing brain organoids with iMacs generated from the same human iPSC line (Park et al., 2023). In such cocultures, iMac differentiated into cells with microglia-like phenotypes and functions. Most importantly, iMac modulated neuronal progenitor cell (NPC) differentiation, limiting NPC proliferation and promoting axonogenesis, profoundly remodelling organoid physiology. Thus, in this study, we hypothesised that iMacs could be directly cultured with iPSC-derived hepatocytes (iHeps) from the same IPSC source to both improve iHep development and maturation as well as impart KC-like identity to the iMacs. Upon development of such a model, we tested its application in detecting immune-mediated responses of seven paradigm compounds. Concurrently, we also cultured iMacs in PHCM for 7 days, taking inspiration from our previous studies as well as others (Tasnim et al., 2019; Cai et al., 2014; Tasnim et al., 2016; Baratta et al., 2009), comparing the transcriptomic effect (Picelli et al., 2014; Dobin et al., 2013; Liao et al., 2014; Harrow et al., 2006; Robinson et al., 2010; Benjamini and Hochberg, 1995) of direct contact against soluble factors in the context of hepatic macrophage specialisation. Our results showed improved in vivo correlation based on the effects on cytokine production when iMac-derived KCs were used. Altogether, this study highlights the effect of direct crosstalk between hepatocytes and macrophages in vitro using iPSCs and provides a new human model to test drug-induced cytokine responses.

## Results

### Optimisation of culture conditions allows survival and function of iHeps and iMacs

**Figure 1.**
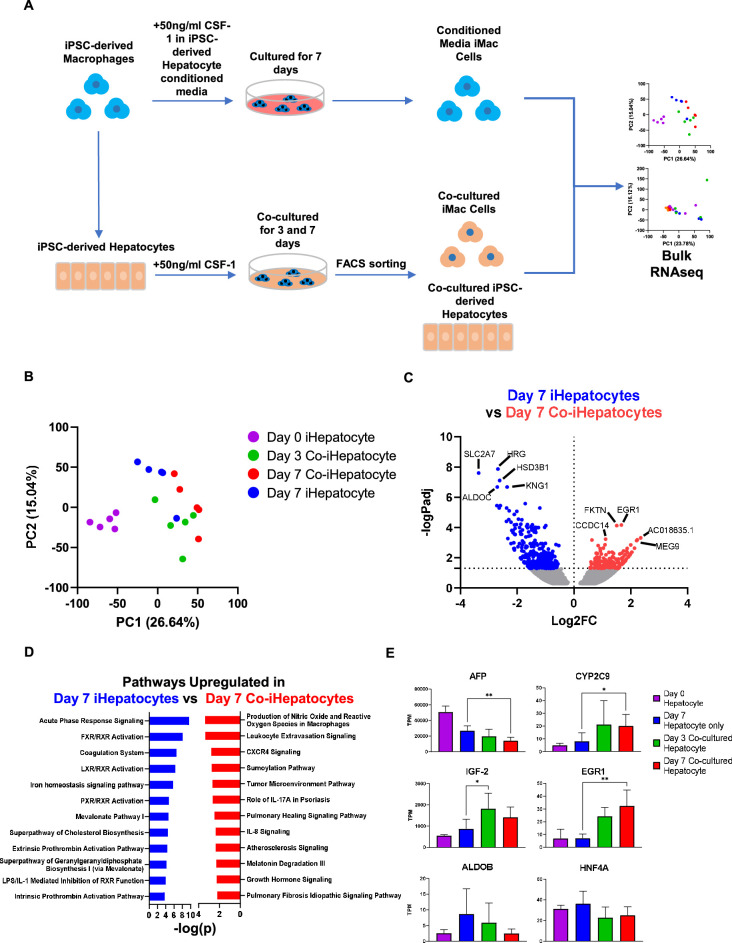
Model development and effects of iMacs on iHeps. (**A**) Schematic of experimental layout. (**B**) Principal component analysis of bulk RNAseq data from Day 0 iHeps, Day 3 co-iHeps, Day 7 co-iHeps, and Day 7 iHeps. (**C**) Volcano plot showing differentially-expressed genes between Day 7 iHeps and Day 7 co-iHeps. (**D**) Differentially expressed upregulated pathways between Day 7 iHeps and Day 7 co-iHeps. (**E**) Gene expression levels of *AFP*, *CYP2C9*, *IGF-2*, *EGR-1*, *ALDOB,* and *HNF4A* in Day 0 iHeps, Day 3 co-iHeps, Day 7 co-iHeps, and Day 7 iHeps. Error bars represented S.D., n=5 independent experiments. Student’s *t*-test was applied. *p<0.05, **p<0.01. Figure 1—source data 1.Differentially expressed genes between the iHeps samples. Figure 1—source data 2.Ingenuity pathway analysis for genes upregulated in Day 7 iHeps vs Day 7 co-iHeps. Figure 1—source data 3.Ingenuity pathway analysis for genes upregulated in Day 7 co-iHeps vs Day 7 iHeps.

In order to investigate the effects of co-culturing iHeps with iMacs, we first established and optimised a co-culture system utilising IMR90 iPSC-derived iHeps and iMacs (Figure 1A). To ensure survival and functionality of both cell types in our desired culture period (at least 1 week), certain key factors had to be accounted for: Hepatocytes are high in metabolic activity and often require frequent media change for replenishment of nutrients and removal of waste. On the other hand, it would be ideal to allow the iHeps and iMacs to interact through direct crosstalk as well as through secreted soluble factors without removing these factors from the system via media exchange. To assess the performance of hepatocytes with more infrequent media changes, expression of key hepatic markers was assessed when media was changed every other day, every 2 days, or every 3 days and compared to a daily media change (Figure 1—figure supplement 1). To compensate for the lower frequency of media change, we tested the effects of 2X supplements; standard media volume (Figure 1—figure supplement 1A) and 2X supplements with 2X standard culture volume (Figure 1—figure supplement 1B). Gene expression was compared as fold change to standard hepatocyte culture conditions (daily media change with 1X supplement and standard media volume). Increasing supplements alone could maintain the expression of ALB (albumin), AAT (alpha-1-antitrypsin), cytochrome P450 (CYP)-1A2, 3A4, 2B6 and UDP-glucuronosyltransferase (UGT)1A3, but not CYP2C19 (36–68% decrease), glutathione S-transferase (GST)A2 (35–66% decrease), and multidrug resistance protein-2 (MRP2; 7–18% decrease) (Figure 1—figure supplement 1A). In contrast, combining the increase in supplements with increase in culture volume resulted in maintenance of all hepatic markers (Figure 1—figure supplement 1B). Albumin and urea production were also maintained to 1–1.4 pg/cell/48 hr and 126–146 pg/cell/48 hr (Figure 1—figure supplement 1C) which is in the range of albumin and urea production reported by us (Tasnim et al., 2019; Tasnim et al., 2016; Tasnim et al., 2015) and others (Rose et al., 2016; Shiraki et al., 2011; Takayama et al., 2013; Xia et al., 2016; Nishimura et al., 2010) previously.

**Figure 1—figure supplement 1.**
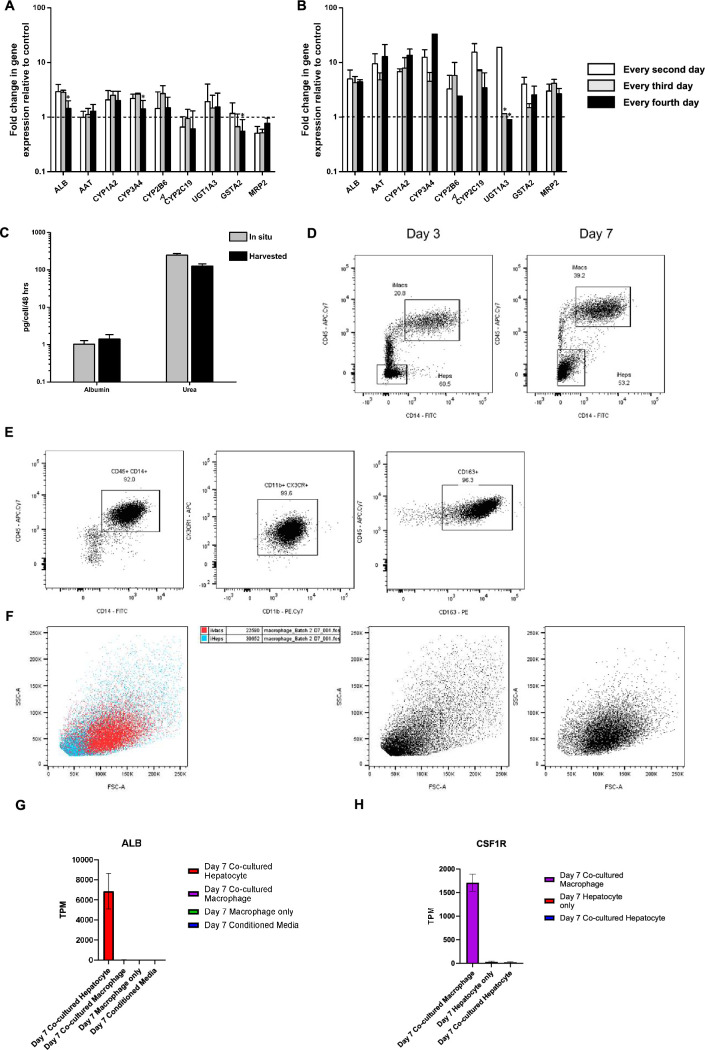
Optimisation of co-culture conditions and validation of purity. (**A, B**) Fold change in expression of key hepatocyte genes in normal media volume and supplement concentration (**A**) or double media volume and supplement concentration (**B**), normalised to normal hepatocyte culture condition. (**C**) Production of albumin and urea in co-culture system after 2 days, normalised to non-co-cultured hepatocytes. (**D**) Flow cytometry of co-culture 3 and 7 days after co-culture. (**E**) Flow cytometry showing purity of macrophages by CD45, CD14, CD11b, CX3CR1, and CD163 before co-culture. (**F**) Flow cytometry showing relative cellular size and granularity of iMac and iHep populations after co-culture. (**G**) Expression of Albumin in Day 7 co-cultured iHeps vs iMacs. (**H**) Expression of CSF1R in Day 7 co-cultured iMacs vs iHeps.

**Figure 1—figure supplement 2.**
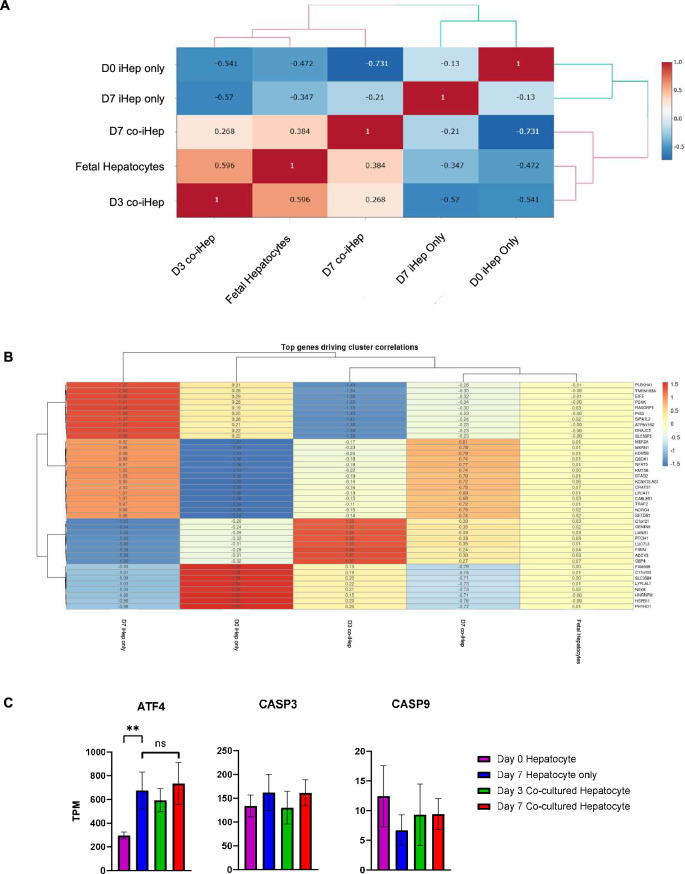
Correlation of iPSC-derived hepatocytes with or without co-culture with iPSC-derived macrophages. (**A**) Pearson correlation of iHeps against fetal hepatocytes from Popescu et al., 2019. (**B**) Top 40 genes influencing Pearson correlation of panel A. (**C**) Expression of ATF4, CASP3, and CASP9 showed no significant upregulation of stress or apoptosis genes after co-culture.

### Co-culture with iMacs improves iHep maturation and development

Cells sorted via flow cytometry on Day 3 and Day 7 (Figure 1—figure supplement 1D and E), and the co-cultured hepatocytes (co-iHeps) and co-cultured iMacs (co-iMacs) were then analysed by RNA sequencing in order to assess if co-culturing leads to transcriptomic changes reflecting improved differentiation and maturation. We also analysed Day 0 and Day 7 mono-culture controls, as well as iMacs cultured in PHCM for 7 days as control groups.

Principal component analysis (PCA) of the iHep samples revealed that although the direction of change was the same for both the mono-cultured and co-cultured iHeps, there was a greater degree of change along the PC1 axis for co-iHeps (Figure 1B). We hypothesised that this might be due to the iMacs promoting further maturation and development of the iHeps, and performed a Pearson correlation between the mono-cultured and co-cultured iHeps against in vivo fetal liver hepatocytes from a published dataset (Popescu et al., 2019), which revealed that the co-cultured iHeps were better correlated with the in vivo hepatocytes than the mono-cultured iHeps (Figure 1—figure supplement 2A). Indeed, looking at the differentially expressed genes (DEGs) between the Day 7 iHeps and Day 7 co-iHeps revealed that the co-iHep upregulated genes associated with tissue morphogenesis and repair like Early Growth Response 1 (*EGR1*) (Pritchard et al., 2010), centrosome duplication such as the long non-coding RNA *CCDC14* (Firat-Karalar et al., 2014), and angiogenesis-related gene like Maternally Expressed Gene 9 (*MEG9*) (Espinosa-Diez et al., 2018; Figure 1C, Figure 1—source data 1). On the other hand, iHeps cultured alone upregulated genes that inhibit proliferation and angiogenesis such as Histone Rich Glycoprotein (*HRG*) (Zhang et al., 2015) and Kinogen 1 (*KNG1*) (Colman et al., 2000; Figure 1C, Figure 1—source data 1). We then performed pathway analysis using Ingenuity and discovered that the top upregulated pathway in Day 7 iHeps was acute phase response signalling, with the associated genes mainly relating to protein production such as Transthyretin (*TTR*) and Serpin Family D Member 1 (*SERPIND1*) (Figure 1—source data 2), while the top upregulated pathway in Day 7 co-iHeps was related to nitric oxide and reaction oxygen species production, consisting mainly of metabolic and phosphatase genes like *c-FOS* and Serine/Threonine protein phosphatase 2A regulatory subunit B” beta (*PPP2R3B*) (Figure 1D, Figure 1—source data 3). A closer look at the pathways upregulated in Day 7 iHeps and Day 7 co-iHeps also revealed that the Day 7 co-iHeps had a stronger tissue development and anabolic signature, upregulating pathways associated with tumour microenvironment, fibrosis and growth hormone signalling, while the Day 7 iHeps upregulated iron and cholesterol pathways.

Finally, we compared the expression of key genes across all the iHep samples (Figure 1E). In line with our hypothesis that co-culturing iMacs with iHeps would improve the maturation of the iHeps, we found that alpha-fetoprotein (*AFP*), a marker that is upregulated during fetal development and then downregulated as hepatocytes mature (Ang et al., 2018) followed a similar trend in our culture system, with lower expression in the Day 7 iHeps as compared to the Day 0 iHeps. Furthermore, the expression levels of *AFP* in Day 3 co-iHeps and Day 7 co-iHeps was even lower than that of Day 7 iHeps. Likewise Hepatocyte Nuclear Factor 4 (*HNFA4*), a transcription factor upregulated in hepatic progenitor cells (Chaudhari et al., 2016), trended towards reduced expression in the co-cultured iHeps, albeit non-significantly. Insulin-like growth factor-2 (*IGF-2*), a key growth factor in fetal development (Agrogiannis et al., 2014), was significantly upregulated in the co-cultured iHeps 3 days after co-culture, with expression diminishing at the 7-day mark. Cytochrome P450 Family 2 Subunit C member 9 (*CYP2C9*), a cytochrome involved in drug metabolism was also significantly upregulated in the co-iHeps, while Aldolase Fructose-Biphosphate B (*ALDOB*), an enzyme whose upregulation is associated with unregulated cell proliferation and poor cancer prognosis (Chang et al., 2018) had a non-significant trend towards downregulation. Taken together, our data suggest that co-culturing the iHeps with iMacs increased their maturation and promotes improved hepatocyte development.

### Co-culture with iHeps imparts KC-like identity to iMacs

**Figure 2.**
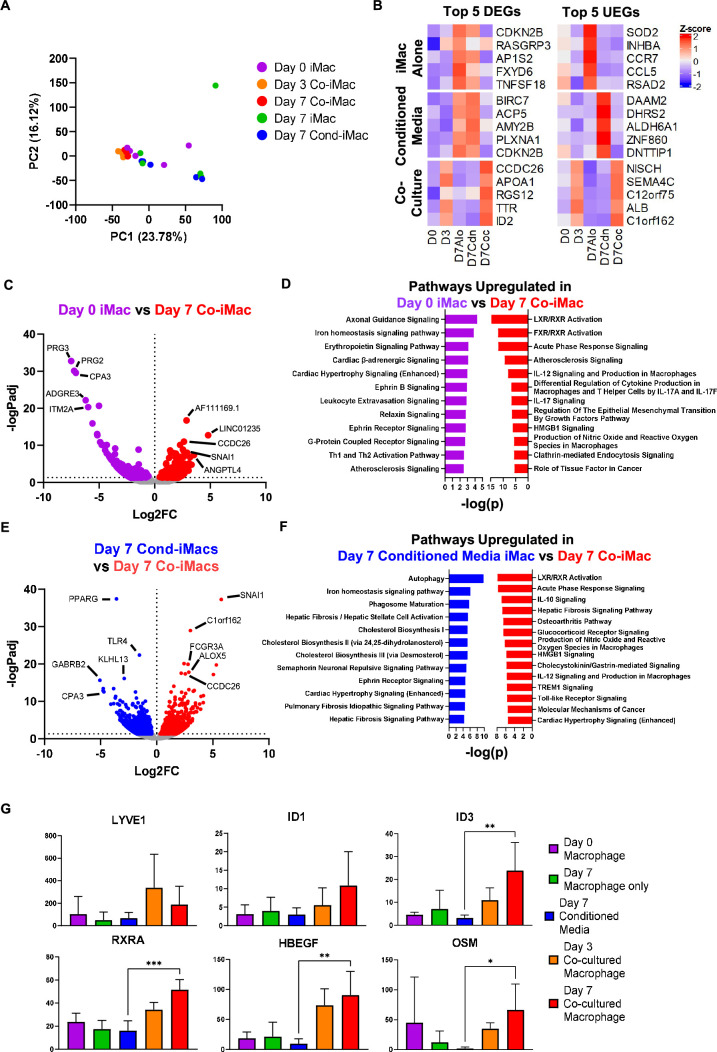
Effects of iHeps on iMacs. (**A**) Principal component analysis of bulk RNAseq data from Day 0 iMacs, Day 3 co-iMacs, Day 7 co-iMacs, Day 7 iMacs, and Day 7 cond-iMacs. (**B**) Heatmap showing the top 5 differentially and uniquely expressed genes from culturing iMacs alone, with conditioned media (cond-iMacs) and after co-culture with iHepatocytes (Day 7 co-iMacs). (**C**) Volcano Plot showing differentially-expressed genes between Day 0 iMacs and Day 7 co-iMacs. (**D**) Differentially expressed upregulated pathways between Day 0 iMacs and Day 7 co-iMacs. (**E**) Volcano plot showing differentially-expressed genes between Day 7 cond-iMacs and Day 7 co-iMacs. (**F**) Differentially expressed upregulated pathways between Day 7 cond-iMacs and Day 7 co-iMacs. (**G**) Gene expression levels of *LYVE1*, *ID1*, *ID3*, *RXRA*, *HBEGF,* and *OSM* in Day 0 iMacs, Day 3 co-iMacs, Day 7 co-iMacs, Day 7 iMacs and Day 7 cond-iMacs. Error bars represented S.D., n=5 independent experiments. Student’s *t*-test was applied. *p<0.05, **p<0.01, ***p<0.001. Figure 2—source data 1.Differentially expressed genes between the iMac samples. Figure 2—source data 2.Ingenuity pathway analysis for genes upregulated in Day 7 co-iMacs vs Day 0 iMacs. Figure 2—source data 3.Ingenuity pathway analysis for genes upregulated in Day 0 iMacs vs Day 7 co-iMacs. Figure 2—source data 4.Ingenuity pathway analysis for genes upregulated in Day 7 cond-iMacs vs Day 7 co-iMacs. Figure 2—source data 5.Ingenuity pathway analysis for genes upregulated in Day 7 co-iMacs vs Day 7 cond-iMacs. Figure 2—source data 6.GO enrichment results for DEGs between Day 7 conditioned media iMacs and embryonic liver macrophages. Figure 2—source data 7.GO enrichment results for DEGs between Day 7 co-cultured iMacs and embryonic liver macrophages.

Next, we turned our attention towards the iMacs within our co-culture system to test if they acquired KC features. PCA analysis showed that the co-iMacs clustered much tighter together, while iMacs that were cultured alone or in hepatocyte conditioned media (cond-iMac) were much more scattered (Figure 2A). This suggests that co-culturing iMacs in direct contact with iHeps may help maintain the identity of the iMacs better, mirroring how ex vivo resident tissue macrophages (RTMs) rapidly lose their identity when removed from their tissue environment (Gosselin et al., 2017). DEG analysis of all the iMac groups showed that both the Day 7 iMacs and the Day 7 cond-iMacs shared an activated and inflammatory profile, upregulating genes such as TNFSF18 (Croft et al., 2012) and ACP5 (Räisänen et al., 2005; Figure 2B, Figure 2—source data 1). Day 7 iMacs had a more migratory profile, uniquely expressing Chemokine Receptor 7 (*CCR7*) and Chemokine Ligand 5 (*CCL5*), while Day 7 cond-iMacs uniquely expressed Dishevelled Associated Activator of Morphogenesis 2 (*DAAM2*), a developmental regulator of the WNT pathway (de Alwis et al., 2021).

**Figure 2—figure supplement 1.**
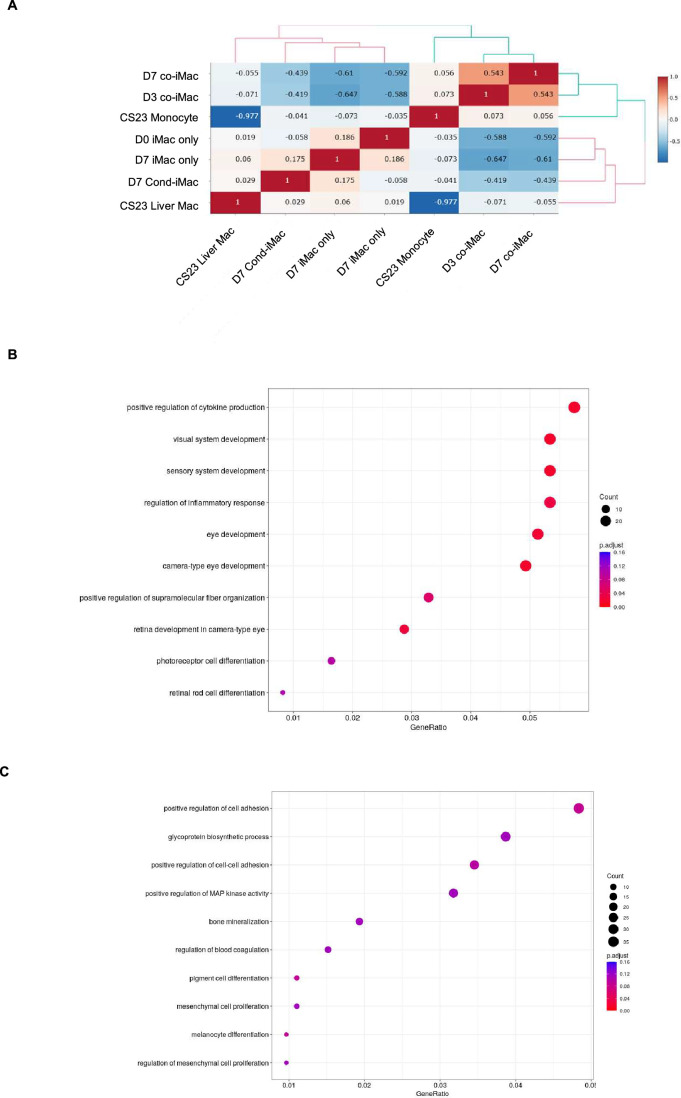
Correlation of iMacs with embryonic liver monocytes and macrophages, and pathway analysis of the DEGs. (**A**) Pearson correlation of iMacs under control, conditioned media or co-culture conditions against embryonic liver monocytes and macrophages from Bian et al., 2020. (**B**) Pathway analysis of DEGs between Day 7 conditioned media iMacs and embryonic liver macrophages. (**C**) Pathway analysis of DEGs between Day 7 co-cultured iMacs and embryonic liver macrophages.

As we were interested in how the presence of iHeps in such co-culture model might educate and impart KC-like identity to the iMacs, we looked at the DEGs between Day 0 iMac and Day 7 co-iMacs. Day 0 iMacs were more immunogenic, expressing Proteoglycan 2 Pro Eosinophil Major Basic Protein 2/3 (*PRG2/PRG3*) and Carboxypeptidase A3 (*CPA3*) (Figure 2C). In contrast, Day 7 co-iMacs upregulated the angiogenic Angiopoietin-like 4 (*ANGPTL4*) (Babapoor-Farrokhran et al., 2015), matching the angiogenic signature observed in the Day 7 co-iHeps. Importantly, Ingenuity pathway analysis revealed that the top pathway in Day 7 co-iMacs is the Liver X Receptor/Farnesoid X Receptor (LXR/RXR) signalling pathway (Figure 2D, Figure 2—source data 5), a key regulator of KC identity (Bonnardel et al., 2019), while the top pathway in Day 0 iMacs is the axonal guidance pathway, consisting of chemokines like C-X-C Motif Chemokine Ligand 12 (*CXCL12*) and C-X-C Motif Chemokine Receptor 4 (*CXCR4*), metalloproteases like ADAM Metallopeptidase with Thrombospondin Type 1 Motif 15 (*ADAMTS15*), and semaphorins like Semaphorin 3C (*SEMA3C*) (Figure 2—source data 6). This suggests that co-culturing iMacs with iHeps initiated a KC-specific programme, while the Day 0 iMacs had a more general and unspecified identity lacking environmental cues. In addition, Day 7 co-iMacs also upregulated pathways associated with tissue repair and remodelling, as well as tissue growth factors, while migratory and immune signalling related pathways were upregulated in the Day 0 iMacs (Figure 2D). Altogether, these data suggest that co-culturing iMacs with iHeps is sufficient to impart a more tissue supportive and KC-like identity to the iMacs.

The use of conditioned media as a surrogate of the in vivo tissue microenvironment is a popular approach, and we wondered how different iMacs cultured in direct co-culture with iHeps would be from iMacs cultured in conditioned media from primary hepatocytes (PHCM). Comparing the DEGs between Day 7 cond-iMacs and Day 7 co-iMacs, the Day 7 cond-iMacs upregulated immune-related genes such as Toll-Like Receptor 4 (*TLR4*) and *CPA3*, while the Day 7 co-iMacs more highly expressed Fc Gamma Receptor IIIa (*FCGR3A*), which is also upregulated in Kupffer Cells (Figure 2E). Pathway analysis showed that the top upregulated pathway in the Day 7 cond-iMacs was autophagy, which might indicate that conditioned media alone was insufficient to maintain proper macrophage biology in the absence of contact with other cells, although some hepatic-related pathways were also upregulated as well (Figure 2F, Figure 2—source data 4). On the other hand, the most highly upregulated pathway in the Day 7 co-iMacs was the FXR/RXR pathway, which along with the upregulation of other anabolic pathways related to hepatic health (Figure 2—source data 5), suggests that co-culturing rather than conditioned media is superior at inducing KC-like identity in macrophages.

We then looked at the expression of selected key KC genes. Lymphatic Vessel Endothelial Hyaluronan Receptor 1 (*LYVE1*), DNA-binding protein inhibitor ID1 (*ID1*) and DNA-binding protein inhibitor ID3 (*ID3*), which are important markers of KC identity (Babapoor-Farrokhran et al., 2015). *ID3* was significantly upregulated in the co-iMacs but not in the cond-iMacs, with a trend towards increased *ID1* and *LYVE1* expression as well (Figure 2G). Retinoid X Receptor Alpha (*RXRA*), the obligate binding partner of LXR (Yue et al., 2005), was also upregulated in only the co-iMacs. Interestingly, we also found that the co-iMacs upregulated the expression of Heparin Binding EGF Like Growth Factor (*HBEGF*) (Dao et al., 2018) and Oncostatin M (*OSM*) (Miyajima et al., 2000), which are potent effectors of liver regeneration and development.

Finally, we compared the iMacs from our study with in vivo embryonic human liver monocytes and KCs (Bian et al., 2020). Pearson correlation revealed that regardless of condition the in vitro derived iMacs were poorly correlated to the in vivo human liver monocyte/macrophages (Figure 2—figure supplement 1), suggesting that either the co-culture duration was insufficient to induce full KC commitment or that there might be missing cues from other cell types that were not present in our co-culture. However, pathway analysis on the DEGs between the embryonic human liver macrophages and either the Day 7 cond-iMacs (Figure 2—figure supplement 1B, Figure 2—source data 6) or Day 7 co-iMacs (Figure 2—figure supplement 1C, Figure 2—source data 7) revealed that pathways relating to cytokine production, sensory development and eye development were differentially regulated between the Day 7 cond-iMacs and embryonic liver macrophages, while there were no significant pathway differences between the Day 7 co-iMacs and embryonic liver macrophages. This suggests that direct co-culture might drive the macrophages to adopt a more tissue developmental or supportive phenotype.

Altogether, the data supports our hypothesis that co-culturing iMacs with iHeps induces a KC-like tissue supportive identity in the iMacs, which in turn promotes the maturation and development of the iHeps.

### Validation of the acquisition of KC-like identity by iMacs and maturation of iHeps in co-culture

**Figure 3.**
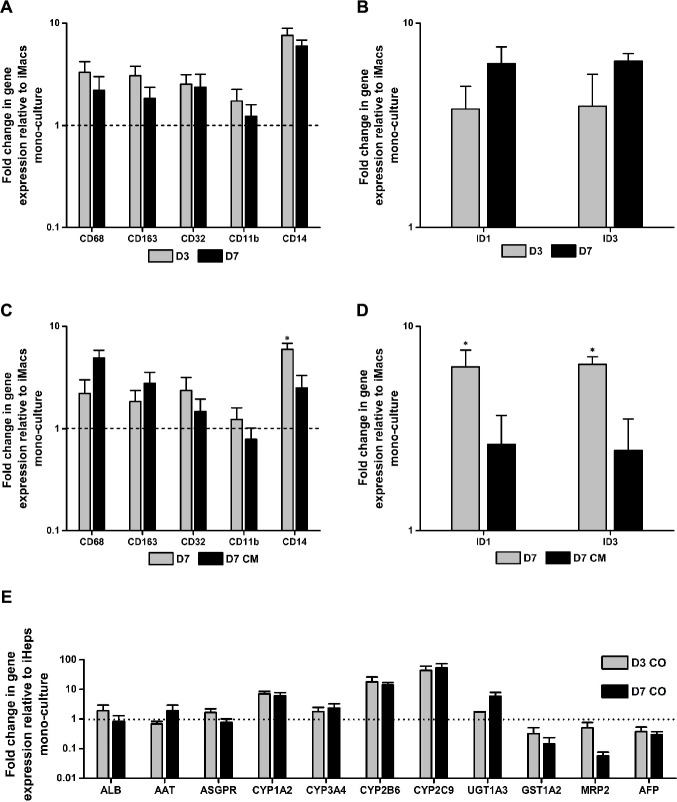
Gene expression of iMac and iHep markers in co-cultures. (**A**) Expression of macrophage markers (left panel) and KC-specific markers (right panel) on Day 3 and Day 7 of co-culture. (**B**) Expression of macrophage markers (left panel) and KC-specific markers (right panel) on Day 7 of co-culture compared to iMac cultured in PHCM. (**C**) Expression of hepatic markers (left panel) on Day 3 and Day 7 of co-culture. Error bars represented S.D., n=5 independent experiments. *p<0.05. Student’s *t*-test was applied. *p<0.05.

We confirmed the observations from the RNA sequencing analysis by analysing gene expression of macrophage and KC-specific markers in Day 3 co-iMacs and Day 7 co-iMacs via RT-PCR. Macrophage markers *CD68*, *CD163*, *CD11b*, *CD32* and *CD14* were all upregulated at Day 3 and Day 7 by 1.8–7.6 fold compared to iMac mono-culture control (Figure 3A). Importantly, KC-specific markers *ID1* and *ID3* were upregulated by 3.8 fold and 3.9 fold respectively at Day 3 and by 6.3 fold and 6.5 fold respectively at Day 7 (Figure 3B). This suggests that macrophage phenotype is maintained during the 7 days of co-culture but KC-specific development increases in a time-dependent manner. When Day 7 co-iMacs were compared to iMacs cultured in PHCM (Day 7 cond-iMacs; without the presence of iHeps), macrophage marker upregulation compared to iMac mono-culture control were similar in both conditions (Figure 3C) but KC-specific *ID1* and *ID3* were upregulated to a lower extent in Day 7 cond-iMacs (2.6 fold and 2.5 fold, respectively) compared to Day 7 co-iMacs (6.3 fold and 6.5 fold, respectively; Figure 3D).

Next, we confirmed the RNA sequence analysis of iHep via RT-PCR. With the exception of Glutathione-S transferase1A2 (*GST1A2*) and Multidrug Resistance Associated Protein 2 (*MRP2*), co-culture with iMacs improved expression of several hepatic marker genes including *ALB*, α–1 antitrypsin (*AAT*), cytochrome P450 enzymes (*CYP1A2*, *CYP3A4*, *CYP2C9*, *CYP2B6*) and UDP-glucuronosyltransferases (*UGT1A3*) by 1.8–53.1 fold at Day 3 and Day 7 of co-culture (Figure 3E). Importantly, *AFP*, a marker that is expressed at high levels in fetal hepatocytes and increases with hepatocyte maturation (Firat-Karalar et al., 2014), had 37% lower expression at Day 3 and 37% lower expression in Day 7 iHeps as compared to Day 0 iHeps (Figure 3E). This is consistent with RNA sequencing data that showed a downregulation of *AFP* (Figure 1E).

Overall, validation of the expression of key markers observed as modulated by RNA sequencing analysis confirms that culturing iMacs with iHeps imparts KC-like phenotype in iMacs and supports maturation and function of the iHeps.

### Co-culture model accurately mimics clinical/in vivo immune responses of drugs

Drug-induced liver injury (DILI) is a complex and critical clinical problem with an estimated incidence rate of up to 19 cases per 100,000 people in western countries (Hoofnagle and Björnsson, 2019). In particular, immune-mediated idiosyncratic drug-induced liver injuries remain especially poorly understood, due in large part to the lack of appropriate human modelling systems (Tasnim et al., 2021). To test the application of the co-culture in detecting immune-mediated response to hepatotoxic drugs, we carefully selected a group of seven drugs based on the following criteria: (1) confirmed reports of immune/inflammation-associated effects according to three databases (Kaplowitz, 2005; Ho et al., 2015; Verma and Kaplowitz, 2009); (2) available clinical data, or in vivo responses if the former is unavailable; and (3) known Cmax (maximum serum concentration of drug) to guide the concentration of drug used in the in vitro model. Drug concentrations used in our system were no more than 20-fold of its Cmax (details of selected drugs are summarised in Figure 4—source data 1; Chen et al., 2016; Thakkar et al., 2020; Xu et al., 2008). The drugs included diclofenac (DIC) (Ademisoye et al., 2018), sulindac (SLD) (Zou et al., 2009), leflunomide (LFM) (Li et al., 2002), amodiaquine (AQ), lamotrigine (LTG) (Tuba Incecayir, 2009), penicillin (PEN), pyrazinamide (PZA) (Bareggi et al., 1987). DIC (Mahdy et al., 2002), SLD (Kang et al., 2001), and LFM (Yao et al., 2004; Manna and Aggarwal, 1999) have been reported to cause a decrease in interleukin-6 (IL-6), whereas AQ and LTG have been reported to cause an increase in IL-6 in patients with drug-induced liver injury (Verma and Kaplowitz, 2009). PEN and PZA were selected as negative controls as no immune-mediated effects of these drugs have been reported. The cultures were co-treated with lipopolysaccharide (LPS), which has been reported as an important initiating co-factor in the development of DILI (Roberts et al., 2007).

**Figure 4.**
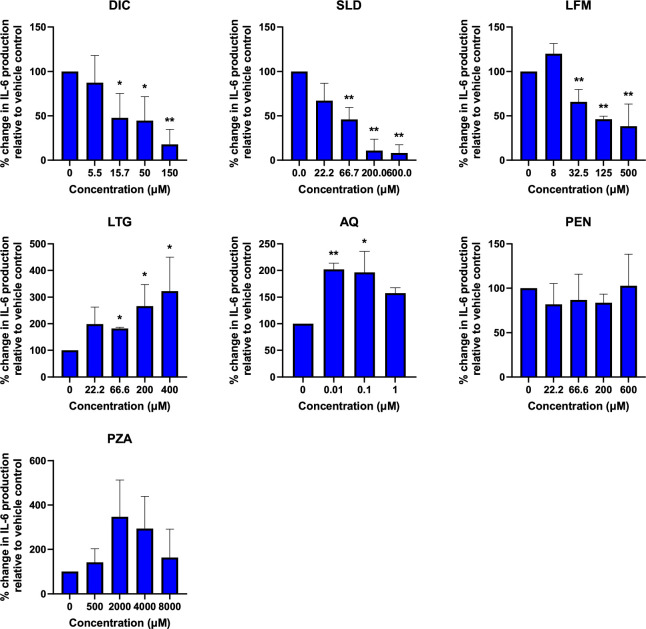
Changes in IL-6 level upon treatment with seven paradigm compounds when iMac-derived KCs were used. Cytokine production was assessed in iMac-derived iKC and iHep co-culture treated with the drug stated in the plot title. Data is expressed as the percentage of the LPS-treated vehicle control. Error bars represented S.D., n=3 independent experiments. One-way ANOVA was applied. *p<0.05, **p<0.01 between treatment and vehicle control. Figure 4—source data 1.Details of drugs used for testing.

**Figure 5.**
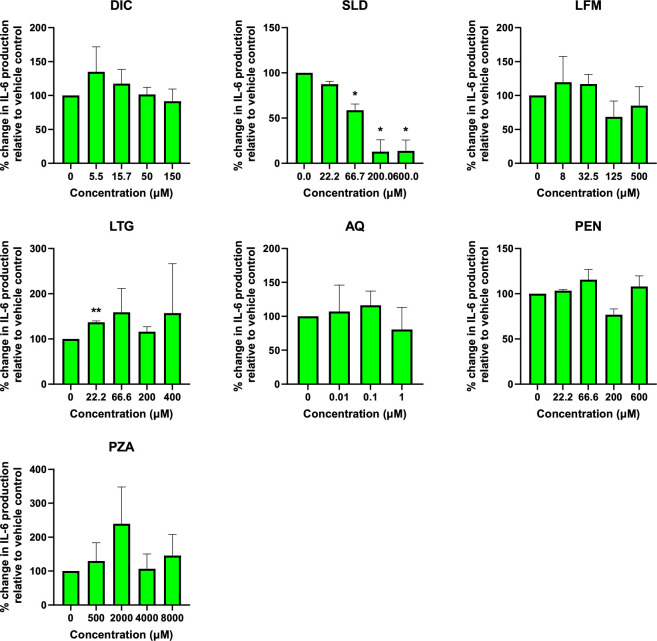
Changes in IL-6 level upon treatment with 7 paradigm compounds without iMac-derived KCs. Cytokine production was assessed in blood monocyte-derived macrophages and iHep co-culture treated with the drug stated in the plot title. Data is expressed as the percentage of the LPS-treated vehicle control. Error bars represented S.D., n=3 independent experiments. One-way ANOVA was applied. *p<0.05, **p<0.01 between treatment and vehicle control.

Our co-culture model could correctly recapitulate IL-6 decrease in DIC (48%, 45%, and 18% at 15.7 μM, 50 μM, and 150 μM, respectively), SLD (46%, 11%, and 8% at 67 μM, 200 μM, and 600 μM, respectively) and LFM (65%, 46%, and 38% at 32.5 μM, 125 μM, and 500 μM, respectively). IL-6 increase upon AQ (157–200 fold) and LTG (155–322 fold) treatment was also correctly recapitulated. No changes in IL-6 level in PEN and PZA-treated samples were observed (Figure 4). Importantly, when iMac-derived iKC-like cells were replaced with blood monocytes/macrophages, none of these cytokine changes were recapitulated with the exception of SLD treatment (Figure 5). This shows, from a functional/application perspective, how iMac-iKCs, but not MoMϕs, are indispensable for in vitro liver models that can detect immune-mediated changes of hepatotoxicants.

In vivo, KCs have been known to release mediators that are early features of inflammatory responses (Steuerwald et al., 2013). They are activated in response to inflammatory stimuli such as bacterial LPS that can damage the liver at large doses in a KC-dependent manner. At smaller doses, LPS activates KCs (without causing liver injury) but sensitises the liver to a variety of other xenobiotics (Roberts et al., 2007). In our model, when the co-culture was co-treated with paradigm hepatotoxicants and LPS, drug-dose-dependent changes in cytokine production were observed (Figure 4), suggesting that the system can indeed mimic physiological responses, demonstrating the importance of liver-specific imprinting of macrophages in recapitulating responses in an in vitro liver model.

## Discussion

IPSC-derived tissue cultures have tremendous potential in their ability to generate cells from all intra-embryonic lineages and have greatly expanded both our knowledge of mammalian tissue development and our technical toolbox for therapy and disease modelling (Rowe and Daley, 2019). However, a tissue is often more than the sum of its parts, and iPSC-derived tissue models have so far proven to be limited in their ability to effectively recapitulate the functionality and complexities of their presumed organs. In our study, we demonstrated how co-culturing iMacs with iHeps leads to not only acquisition of resident tissue characteristics within the iMac population, but also increased maturation of iHeps. We demonstrated that iPSC-derived Heps can directly impart hepatic macrophage-like identity to iMacs, giving them an iKC-like phenotype and genotype without the need for any additional soluble factors or components present in conditioned media. Application of this model was demonstrated by the ability of the system to mimic clinical IL-6 responses of five paradigm immune-mediated hepatotoxicants. Two additional hepatotoxicants with no known immune-mediated responses did not show any response in our model, demonstrating the specificity of the system. Finally, when the immune-mediated drug effects were tested in a system with iHeps and PBMC-derived macrophages, expected cytokine responses were not observed, indicating the importance of iKC-iHep co-culture in predicting drug mediated-immune responses.

Animal models have been used to model immune-mediated liver injury (Kuna et al., 2018; Shenton et al., 2004; Ng et al., 2012); however, consistent liver injury was not observed upon treatment with drugs known to cause immune response in humans, even at high doses (Uetrecht, 2019). In cases where injury was observed, the characteristics were different from humans and possibly manifested through different mechanisms (Ng et al., 2012). Such interspecies variations, contradictory findings as well as cost and ethical issues have spurred the development of in vitro models to supplement in vivo animal models. In vitro models, possibly due to the lack of appropriate human immune cell sources, have focused on soluble factor-driven single hepatocyte cultures in combination with inflammatory mediators or animal cell co-cultures (Tasnim et al., 2021). However, mono-cultures lack cell–cell interactions, and animal in vitro models often fail to recapitulate in vivo and clinical findings. In vitro hepatocyte-KC models have previously been used; however, most of them have utilised animal cells (Bonzo et al., 2015; Tukov et al., 2006) which often prove inadequate in recapitulating in vivo or clinical findings. In order to avoid interspecies variations and improve the predictivity of in vitro models, appropriate human models still need to be developed and characterised.

The main challenge in studying human KCs in in vitro models is the cell source. The source of commercial primary human KCs (PhKCs) is limited and costly and high donor-to-donor variability is often observed (Kegel et al., 2015). Alternative human cell sources include THP-1 cells and peripheral monocyte-derived macrophages; however their cytokine profiles are dramatically different from human KCs (Shaw et al., 2007; Kermanizadeh et al., 2019) and they lack liver-specific imprinting (Bonnardel et al., 2019). Even when human KCs were used, the work mostly focused on co-culture dependent improvement of hepatocyte function (Tasnim et al., 2021; Novik et al., 2010), rather than interaction between the two cell-types and immune-mediated effect of drugs. Moreover, these models are often non-isogenic, which further confounds the inflammatory readouts. Hence, in our study, we aimed to first determine if the presence of hepatocytes could impart a tissue-resident-like phenotype to iMacs and drive them to be more Kupffer cell-like, then measured the systemic response to drug treatment compared to using monocyte-derived macrophages. While all tested drugs showed expected changes in cytokine production in the iMac-iHep co-culture (Figure 4), these changes were not recapitulated when the iMacs were replaced by MoMϕs (Figure 5) with the exception of SLD. Interestingly, expected IL-6 increase upon treatment with LFM could not be recapitulated in our previous model of iHep-iKCs co-culture where iKCs were generated using conditioned media from primary hepatocytes (data not shown). However, in our current model, LFM treatment resulted in the expected increase in IL-6, suggesting the potential importance of direct cellular cross-talk in iKCs differentiation and function.

Development of a suitable model system is further complicated by the understanding that KCs are derived from embryonic macrophages that seed the liver early on during development (Mass et al., 2016; Lee and Ginhoux, 2022; Scott et al., 2016). This also implies that conventionally used human peripheral blood MoMϕs might not be able to fully mimic KC biology. Thankfully, iMacs more closely resemble embryonic macrophages than MoMϕs (Takata et al., 2017) and as demonstrated in our own experiments, were able to acquire features of KCs upon co-culture with iHeps. Soluble factors were only partially responsible for this tissue adaptation, as in contrast to the iMacs that were cultured in hepatocyte conditioned media, the co-cultured iMacs upregulated genes in the LXR/RXR pathway, which has been shown to be important for KC specification and identity (Bonnardel et al., 2019), further emphasising the importance of cell–cell contact for the acquisition of RTM identity. Furthermore, we also observed that these co-cultured iMacs were upregulating pathways associated with tissue repair and development, which might explain the increased maturity of the iHeps within the co-culture system. This is particularly relevant not only for hepatic model systems, but for other iPSC-derived model systems in general, as one of the key limitations of these systems is the functional and phenotypic immaturity of the differentiated cells (Doss and Sachinidis, 2019). Co-culturing iMacs with these immature iPSC-derived tissue model systems might provide the missing developmental cues needed for more physiologically and functionally relevant patient and disease models as well.

However, the results presented in our study are not without their own limitations as well. We primarily utilised bulk sequencing data in our analysis, and although we have demonstrated that there is minimal contamination between the hepatocyte and macrophage compartments of our co-culture, we are unable to completely rule out the minor possibility. Furthermore, our co-culture model is limited to only hepatocytes and macrophages, and might not fully address the complexity of the liver microenvironment. For instance, our model does not include other important cell types such as hepatic stellate cells (Martínez García de la Torre et al., 2025) or sinusoidal endothelium cells (Gage et al., 2020). This could perhaps explain why the Day 3 co-cultured iHeps showed greater similarity to their in vivo counterparts than the Day 7 co-cultured iHeps, as the two-cell type co-culture alone might be enough to initiate co-development but insufficient to maintain it. Indeed, we observed transient upregulation of *IGF2* in the Day 3 co-cultured iHeps (Figure 1E), suggesting the activation of a fetal developmental program. We are still unable to determine if its transient nature is an inherent feature of liver development or due to insufficient signalling. Finally, we also only specifically looked at *IL-6* expression as a systemic readout of liver function within our co-culture system. Although we were able to recapitulate the differential drug-specific expression of *IL-6* observed in various in vivo animal models of DILI (Kaplowitz, 2005; Ho et al., 2015; Verma and Kaplowitz, 2009; Mahdy et al., 2002; Kang et al., 2001; Yao et al., 2004; Manna and Aggarwal, 1999), we did not explore the mechanisms behind the individual drug responses. It is important to note that a simplistic two-cell co-culture model system is still insufficient to fully encompass the complex systemic, metabolic and genetic landscape of a patient-specific condition such as DILI, but should instead serve to highlight the importance of incorporating other secondary cell types into model systems for a more holistic and accurate representation of tissue activity. Nevertheless, we believe that the development of co-culture systems containing multiple distinct cell types is an exciting next step in furthering our understanding of proper tissue specification and maturation, not only as a model of development but also tissue function.

## Materials and methods

### Maintenance of iPSCs

Human IMR90-iPSCs were obtained from WiCell Research Institute (Madison, WI). The cells were maintained under antibiotic-free conditions in mTESR media (StemCell Technologies, 85850) on Matrigel (Corning, 354234)-coated plates. Cells were passaged using ReLESR (StemCell Technologies, 05872) or Dispase (StemCell Technologies, 07913) as per manufacturer’s protocol whenever they reached 80% confluency.

### Differentiation of iPSCs to iHeps

IMR90-iPSCs were differentiated into iPSC-hepatocytes (iHeps) as previously described (Cai et al., 2014). Briefly, the iPSCs were cultured to 80% confluency, then made into single cells using Cell Dissociation Buffer (GIBCO, 13151014) and seeded at 1.5 × 10^5^ cells/cm^2^ on the desired well plate format (i.e. 1.5 × 10^6^ cells for a 6-well plate) and placed under hypoxic conditions (5% O_2_ and 5% CO_2_). Growth factors and media used starting from the next day to Day 20 are detailed in Tasnim et al., 2019. Media was changed every day and hypoxic conditions were used during days 5–15 of differentiation. After 20 days of differentiation, iPSC-derived macrophages (iMacs) were either directly added to the iHeps as described in the main text or iHeps were harvested for seeding into smaller well formats for drug testing. The harvesting protocol has been previously optimised by us and has been detailed in Tasnim et al., 2016.

### Differentiation of iPSCs to iMacs

IMR90-iPSCs were differentiated into iMacs as previously described (Takata et al., 2017). Briefly, the iPSCs were cultured to 80% confluency, then passaged using ReLESR. As ReLESR releases the cells as small cell clumps, the passage conditions were optimised to result in roughly 1 × 10^5^ cells in each well of a 6-well plate (Day –1). Starting from the next day (Day 0) to Day 16, the cells were cultured in Stempro Media, consisting of Stempro-34 SFM (GIBCO, 10639-011), supplemented with 200 ug/mL Human Transferrin (Roche, 10-652-202-001), 1x L-Glut, 1x Pen/Strep, 0.5 mM Ascorbic Acid (Sigma, A4544), and 0.45 mM MTG (Sigma, M6145). A full media change was done every other day for the next 16 days, supplemented with the following cytokines: Differentiation Day 0 (5 ng/mL BMP4, 50 ng/mL VEGF, and 2 uM CHIR99021), Differentiation Day 2 (5 ng/mL BMP4, 50 ng/mL VEGF, and 20 ng/mL FGF2), Differentiation Day 4 (15 ng/mL VEGF and 5 ng/mL FGF2), Differentiation Day 6–10 (10 ng/mL VEGF, 10 ng/mL FGF2, 50 ng/mL SCF, 30 ng/mL DKK-1 (RnD, 5439-DK), 10 ng/mL IL-6 (RnD, 206-IL), and 20 ng/mL IL-3), Differentiation Day 12 and 14 (10 ng/mL FGF2, 50 ng/mL SCF, 10 ng/mL IL-6, and 20 ng/mL IL-3). From Day 16, the cells were cultured in 75% IMDM with GlutaMAX (GIBCO, 31980-030), 25% F12 (GIBCO, 11765-047), 1x N2 supplement (GIBCO, 17502-048), 1x B27 Supplement (GIBCO, 17504-001), 0.05% BSA (GE Healthcare, SH30574), and 1x Pen/Strep, supplemented with 50 ng/mL of CSF-1 (RnD, 216-MC), with a full media change every 3 days. In addition, for the first 8 days of differentiation, the cells were kept in a hypoxic incubator (5% O_2_ and 5% CO_2_), before being transferred to a standard cell culture incubator for the next 18 days. After transfer to a standard cell culture incubator, floating cells were collected every media change and resuspended back into the culture to retain the hematopoietic progenitors. After 26 days, the floating cells were collected, and the purity of the iMacs was determined by flow cytometry (Supp Fig 1E).

### Differentiation of human peripheral blood monocytes (PBMCs) into monocyte-derived macrophages

Human PBMCs were purified from blood apheresis cones by ficoll gradient (Cytiva, 17-1440-02), before being magnetically sorted with CD14+ microbeads (Miltenyi, 130-050-021). 1 × 10^6^ CD14+ cells were then seeded per well of a 6-well plate in RPMI 1640 (Hyclone, SH30027.01) with 10% FBS (Biowest, S1810-500), 1x Pen/Strep, 1x Sodium Pyruvate, 1x NEAA, and 1x L-Glut with 50 ng/mL CSF-1 and cultured for 7 days. The purity of the monocyte-derived macrophages was then determined by flow cytometry.

### Co-culture of iMacs and monocyte-derived macrophages with iHeps

2 × 10^5^ iMacs or monocyte-derived macrophages were added to 5 × 10^5^ iHeps during a full media change (4 mL) per well of a 6-well plate on Day 0. We chose this ratio as it has been previously described that in the adult mouse liver, Kupffer cells can be up to 35% of hepatocyte numbers (Baratta et al., 2009). This media was comprised of Williams’ Medium E containing the following supplements: Penicillin/Streptomycin (10,000 U/mL/(10,000 μg/mL) – final conc = 1%, ITS+ - final conc = 2%, 4 mM) GlutaMAX, 30 mM HEPES buffer. The concentrations of the supplements were double that of standard conditions used due to the infrequent media change (please see section 3.1 for more details). In addition, 50 ng/mL of CSF-1 was added to each well. The cells were cultured for 7 days, with a half-media change (2 mL) on Day 4 consisting of the same media as described above with 100 ng/mL of CSF-1, resulting in a final concentration of 50 ng/mL CSF-1 in the well. The cells were collected by digesting with Accutase (StemCell, 07920) for flow cytometry, RNAseq analysis, and qPCR. For pharmacological testing, the number of iHeps and iMacs were downscaled to 96-well plates while maintaining the same iHep:iMac ratio (2.5:1).

### Flow cytometry

Standard staining procedures were used to prepare the cells for flow cytometry analysis. Briefly, cells were dissociated into single cells using the different methods described above for the various tissues, before being incubated with 100 uL of antibody containing FACS buffer (1% BSA and 4 mM EDTA in PBS) per 5 million cells for 20 minutes at 4°C. The cell suspension was then washed with 5 mL of FACS buffer, centrifuged at 400 × *g*, and the supernatant was removed. The cells were finally resuspended in PBS containing 3 uM DAPI (Invitrogen, D1306). Data was acquired by LSRII (BD Bioscience) and analysed by Flow Jo (Tree Star, Inc). For cell sorting, cells were sorted using FACS Aria II (BD Bioscience) or FACS Aria III (BD Bioscience). The following antibodies were used: FITC-conjugated anti-human CD14 (Biolegend, 325604), APC/Cy7-conjugated anti-human CD45 (Biolegend, 368516), PE-conjugated anti-human CD163 (R&D Systems, FAB1607P-100), Alexa Fluor 647-conjugated anti-human CX3CR1 (Biolegend, 341608), and PE/Cy7-conjugated anti-human CD11b (eBioscience, 25-0118-42).

### Bulk RNA-seq

Total RNA was extracted using Arcturus PicoPure RNA Isolation kit (Thermo Fisher, KIT0204) according to the manufacturer’s protocol. All RNAs were analysed on Labchip GX system (PerkinElmer, USA) for quality assessment with median RNA Quality Score of 9.15. cDNA libraries were prepared using 2 ng of total RNA and 1 uL of a 1:50,000 dilution of Ambion ERCC RNA Spike-in Controls (Thermo Fisher, 4456740) using the SMARTSeq v2 protocol described (Picelli et al., 2014) with the following modifications: (1) use of 20 mM TSO and (2) use of 250 pg of cDNA with 1/5 reaction of Nextera XT kit (Illumina, FC-131). The length distribution of the cDNA libraries was monitored using DNA High Sensitivity Reagent Kit (PerkinElmer, CLS60672) on the Labchip GX system (PerkinElmer). All samples were subjected to an indexed PE sequencing run of 2 × 51 cycles on HiSeq 2000 (Illumina) at 16 samples per lane.

### Bulk RNA-seq analysis

Paired-end raw reads were mapped to GRCh38 human genome build using STAR aligner (Dobin et al., 2013). Genes were counted for reads that were mapped to genes using featureCounts (Liao et al., 2014) based on GENCODEv29 gene annotation (Harrow et al., 2006). Log2 transformed counts per million mapped read (log2CPM) and log2 transformed reads per kilobase per million mapped reads (log2RPKM) were computed using edgeR Bioconductor package (Robinson et al., 2010). Across all samples, genes with log2CPM inter-quartile range (IQR) less than 0.5 were filtered out from subsequent differential expression gene (DEG) analysis. DEG analyses for culture condition comparisons were all done using edgeR. Selection of DEGs was done with Benjamini–Hochberg (Benjamini and Hochberg, 1995) which adjusted p-values<0.05. R function ‘prcomp’ was used to perform PCA on log2RPKM values. The pseudobulk values were calculated using the ‘AverageExpression’ function in Seurat. The Pearson correlation was calculated using the ‘cor’ function in R. The GO enrichment was calculated using the ‘enrichGO‘ function from the ClusterProfiler package.

### Quantitative real-time PCR (qPCR)

Total RNA extracted using RNeasy Plus Micro-kit (Qiagen, 74034) was quantified using a NanoDrop ND-1000 Spectrophotometer and converted to cDNA using iScript cDNA synthesis kit (Bio-Rad Laboratories, 1708890). qPCR was performed in 7000 Fast Real-Time PCR System (Applied Biosystems, Foster City, USA) with FastStart Universal SYBR Green Master (Rox) (Roche, 04 913 850 001) and primers from GeneCopoeia, Inc (Rockville, MD, USA). Glyceraldehyde-3-phosphate dehydrogenase (GAPDH) served as internal control. Accession numbers of tested genes are listed in the table below:

**Table:** 

Target gene	Gene name	Accession
GAPDH	glyceraldehyde-3-phosphate dehydrogenase	NM_00156799.2
AFP	Alpha fetoprotein	NM_000295
AAT	Alpha-1 antitrypsin	NM_001134
ASGPR	Asialoglycoprotein receptor	NM_001671
ALB	Albumin	NM_000477.3
CYP1A2	Cytochrome P450 1A2	NM_000761.5
CYP3A4	Cytochrome P450 3A4	NM_017460.5
CYP2B6	Cytochrome P450 2B6	NM_000767.4
CYP2C9	Cytochrome P450 2C9	NM_000771.3
UGT1A3	UDP-glucuroosyltransferase 1A3	NM_019093.2
GST1A2	Glutathione S-Transferase	NM_000846.3
MRP2	Multidrug resistance-associated protein 2	NM_000392
CD14	Cluster of differentiation 14	NM_001174105.1
CD32	Cluster of differentiation 32	NM_001136219.1
CD68	Cluster of differentiation 68	NM_001251.2
CD163	Cluster of differentiation 163	NM_203416.3
ID1	Inhibitor of DNA-binding protein 1	NM_002165.3
ID3	Inhibitor of DNA-binding protein 3	NM_002167.4

### Enzyme-linked immunosorbent assay (ELISA) for measurement of cytokines

Interleukin-6 (IL-6) levels in the media were measured using human IL-6 ELISA kit (Abcam, ab178013) according to the manufacturer’s instructions. The cytokine production from dosed cells was normalised with the viability of cells measured from the same well. This ensures that changes in cytokines are not due to changes in cell numbers that might arise upon drug treatment. The normalised cytokine level was expressed as percentage of DMSO +LPS which allowed better comparison between different batches of experiments. This normalisation approach has been used in previous studies (Tasnim et al., 2015).

### Cell viability assay

Cell viability was examined with AlamarBlue cell viability assay (Thermo Fisher Scientific Inc, Dal1025) according to manufacturer’s instructions. Briefly, AlamarBlue was diluted 10-fold using PBS containing 2 mg/mL of glucose and this working solution was added to the cells and incubated for 1 hr. Fluorescence (Ex: 530 nm, Em: 590 nm) readings were obtained using Tecan Microplate Reader M1000 PRO.

### Drug administration

Following set up of co-culture as described in 2.5, the model was subjected to treatments including 100 ng/mL LPS (Sigma-Aldrich, L2654), and drugs (Sigma-Aldrich) (with/without LPS) for 48 hr. Stock solutions of drugs were prepared in dimethyl sulfoxide (DMSO) and diluted in medium prior to use. Medium containing 0.1% DMSO was used as vehicle control except. After 48 hr, supernatant was collected for cytokine measurement and cell viability in the same wells were measured as described in 2.11.

### Statistical analysis

Mean and standard deviation were obtained from at least three independent batches of cells. Unpaired, paired Student’s *t*-test and one-way or two-way analysis of variance (ANOVA) were performed accordingly at an overall confidence level of 95% using Prism software (GraphPad, San Diego, CA, USA) and indicated below each figure.

## Data Availability

Raw data from RNA-seq analysis have been deposited in the NCBI Gene Expression Omnibus under accession number GSE307755. The following dataset was generated: LeeCZW TasnimF SethiR ChenJ YuH 2025Direct contact between iPSC-derived macrophages and hepatocytes drives reciprocal acquisition of Kupffer cell identity and hepatocyte maturationNCBI Gene Expression OmnibusGSE30775510.7554/eLife.108938PMC1331368742371995 The following previously published datasets were used: PolanskiK HaniffaM StephensonE PopescuDM 2019Human fetal liver, skin and kidney single cell transcriptome dataE-MTAB-7407ArrayExpress BianZ HuangT GongY LeeCZW ShiH 2020Deciphering human macrophage development at single-cell resolutionNCBI Gene Expression OmnibusGSE13334510.1038/s41586-020-2316-732499656

## Additional files

MDAR checklist
