# Supplementary material for: Direct contact between iPSC-derived macrophages and hepatocytes drives reciprocal acquisition of Kupffer cell identity and hepatocyte maturation
Source: eLife. 2026 Jun 29;14:RP108938. doi: 10.7554/eLife.108938 (PMC13313687; doi:10.7554/eLife.108938)
Supplement: Peer review history

# Editor report: eLife Assessment

**DOI:** 10.7554/eLife.108938.3.sa0

**Authors:** Simon Yona

**Affiliations:** The Hebrew University of Jerusalem Israel

**Keywords:** Convincing, Fundamental

This timely and **fundamental** study presents an innovative iPSC based co-culture system to model Kupffer cell–hepatocyte interactions and hepatotoxicity, demonstrating reciprocal acquisition of tissue identity and enhanced hepatocyte maturation. The work is **convincing**, supported by well-executed methodology and functional validation, including physiologically relevant, concentration-dependent hepatotoxic responses. The research approach is promising and of broad interest, further clarification of experimental design and interpretation may strengthen its impact.

# Referee report: Reviewer #1 (Public review)

**DOI:** 10.7554/eLife.108938.3.sa1

The manuscript presents a compelling new in vitro system based on isogenic co-cultures of human iPSC-derived hepatocytes and macrophages, enabling the modelling of hepatic immune responses with unprecedented physiological relevance. The authors show that co-culture leads to enhanced maturation of hepatocytes and tissue-resident macrophage identity, which cannot be achieved through conditioned media alone. Using this system, they functionally validate immune-driven hepatotoxic responses to a panel of drugs and compare the system's predictive power to that of monocyte-derived macrophages. The results underscore the necessity of macrophage-hepatocyte crosstalk for accurate modelling of liver inflammation and drug toxicity in vitro. The manuscript is clearly written and addresses a key limitation in liver organoid systems: the lack of immune complexity and tissue-specific macrophage imprinting.

Strengths:

• Novelty and Relevance: The study presents a highly innovative co-culture system based on isogenic human iPSCs, addressing an unmet need in modelling immune-mediated hepatotoxicity.

• Mechanistic Insight: The reciprocal reprogramming between iHeps and iMacs, including induction of KC-specific pathways and hepatocyte maturation markers, is convincingly demonstrated.

• Functional Readouts: The application of the model to detect IL-6 responses to hepatotoxic compounds enhances its translational relevance.

Weaknesses:

The co-culture model with monocyte-derived macrophages is not fully characterised, making comparisons less informative.

# Referee report: Reviewer #3 (Public review)

**DOI:** 10.7554/eLife.108938.3.sa2

Summary:

In this study, the authors establish a human in vitro liver model by co-culturing induced hepatocyte-like cells (iHEPs) with induced macrophages (iMACs). Through flow cytometry-based sorting of cell populations at days 3 and 7 of co-culture, followed by bulk RNA sequencing, they demonstrate that bidirectional interactions between these two cell types drive functional maturation. Specifically, the presence of iMACs accelerates the hepatic maturation program of iHEPs, while contact-dependent cues from iHEPs enhance the acquisition of Kupffer cell identity in iMACs, indicating that direct cell-cell interactions are critical for establishing tissue-resident macrophage characteristics.

Functionally, the authors show that iMAC-derived Kupffer-like cells respond to pathological stimuli by producing interleukin-6 (IL-6), a hallmark cytokine of hepatic immune activation. When exposed to a panel of clinically relevant hepatotoxic drugs, the co-culture system exhibited concentration-dependent modulation of IL-6 secretion consistent with reported drug-induced liver injury (DILI) phenotypes. Notably, this response was absent when hepatocytes were co-cultured with monocyte-derived macrophages from peripheral blood, underscoring the liver-specific phenotype and functional relevance of the iMAC-derived Kupffer-like cells. Collectively, the study proposes this co-culture platform as a more physiologically relevant model for interrogating macrophage-hepatocyte crosstalk and assessing immune-mediated hepatotoxicity in vitro.

Strengths:

A major strength of this study lies in its systematic dissection of cell-cell interactions within the co-culture system. By isolating each cell type following co-culture and performing comprehensive transcriptomic analyses, the authors provide direct evidence of bidirectional crosstalk between iMACs and iHEPs. The comparison with single-culture controls is particularly valuable, as it clearly demonstrates how co-culture enhances functional maturation and lineage-specific gene expression in both cell types. This approach allows for a more mechanistic understanding of how hepatocyte-macrophage interactions contribute to the acquisition of tissue-specific phenotypes

Weaknesses:

(1) Overreliance on bulk RNA-seq data:

The primary evidence supporting cell maturation is derived from bulk RNA sequencing, which has inherent limitations in resolving heterogeneous cellular states and functional maturation. The conclusions regarding hepatocyte maturation are based largely on increased expression of a subset of CYP genes and decreased AFP levels - markers that, while suggestive, are insufficient on their own to substantiate functional maturation. Additional phenotypic or functional assays (e.g., metabolic activity, protein-level validation) would significantly strengthen these claims.

(2) Insufficient characterization of input cell populations:

The manuscript lacks adequate validation of the cellular identities prior to co-culture. Although the authors reference previously published protocols for generating iHEPs and iMACs, it remains unclear whether the cells used in this study faithfully retain expected lineage characteristics. For example, hepatocyte preparations should be characterized by flow cytometry for ALB and AFP expression, while iMACs should be assessed for canonical macrophage markers such as CD45, CD11b, and CD14 before co-culture. Without these baseline data, it is difficult to interpret the magnitude or significance of any co-culture-induced changes.

(3) Quantitative assessment of IL-6 production is insufficient:

The analysis of drug-induced IL-6 responses is based primarily on relative changes compared to control conditions. However, percentage changes alone are inadequate to capture the biological relevance of these responses. Absolute cytokine production levels - particularly in response to LPS stimulation - should be reported and directly compared to PBMC-derived macrophages to determine whether iMAC-derived Kupffer-like cells exhibit enhanced cytokine output. Moreover, the Methods section should clearly describe how ELISA results were normalized or corrected to account for potential differences in cell number, viability, or culture conditions.

(4) Unclear mechanistic interpretation of IL-6 modulation:

The observed changes in IL-6 production upon drug treatment cannot be interpreted solely as evidence of Kupffer cell-specific functionality. For instance, IL-6 suppression by NSAIDs such as diclofenac is well known to result from altered prostaglandin synthesis due to COX inhibition, while leflunomide's effects are linked to metabolite-induced modulation of immune cell proliferation and broader cytokine networks. These mechanisms are distinct from Kupffer cell identity and may not directly reflect liver-specific macrophage function. Consequently, changes in IL-6 secretion alone - particularly without additional mechanistic evidence or analysis of other cytokines - are insufficient to conclude that co-culture with hepatocytes drives the acquisition of bona fide Kupffer cell maturity.

Reviewers comments to revised manuscript.

The authors successfully established an isogenic, iPSC-derived human liver co-culture model to investigate the role of hepatocyte-macrophage interactions in driving Kupffer cell (KC) identity and hepatocyte maturation. By utilizing a single genetic background, the authors effectively minimized the experimental variability often encountered in non-isogenic systems. A significant highlight of this work is the demonstration that direct co-culture-as opposed to conditioned media alone-is a primary driver for critical KC identity markers such as ID1 and ID3. Furthermore, the model's ability to recapitulate complex clinical IL-6 responses to known hepatotoxicants where standard models have failed underscores its potential utility for early-stage DILI screening. However, there are significant methodological concerns regarding the data analysis. While the study compares four or five distinct experimental groups (e.g., Day 0, Day 7, Day 3 co-culture, and Day 7 co-culture), the authors utilized Student's t-tests for these comparisons. This approach does not account for the multiple comparisons problem and increases the risk of Type I errors. Additionally, while IL-6 secretion is used as a primary functional readout, the individual mechanisms behind these drug responses were not explored experimentally. Finally, Pearson correlation analysis indicates that the iMacs remain poorly correlated with actual in vivo human embryonic liver macrophages, suggesting that the "imprinting" of true KC identity remains incomplete.

# Author comment: Author response

**DOI:** 10.7554/eLife.108938.3.sa3

**Authors:** Christopher Zhe Wei Lee, Farah Tasnim, Xiaozhong Huang, Raman Sethi, Yoohyun Song, Tatsuya Kozaki, Sebastiaan De Schepper, Nicholas Ang, Ivy Low, You Yi Hwang, Jinmiao Chen, Hanry Yu, Florent Ginhoux

**Affiliations:** Singapore Immunology Network, A*STAR Singapore Singapore; A*STAR Singapore Singapore; A*STAR Singapore Singapore; Bioinformatics Institute (BII), A*STAR Singapore Singapore; Institute of Bioengineering and Bioimaging, A*STAR Singapore Singapore; Singapore Immunology Network, A*STAR Singapore Singapore; Singapore Immunology Network, A*STAR Singapore Singapore; Singapore Immunology Network, A*STAR Singapore Singapore; Singapore Immunology Network, A*STAR Singapore Singapore; Singapore Immunology Network, A*STAR Singapore Singapore; Singapore Immunology Network, A*STAR Singapore Singapore; Singapore Immunology Network Singapore Singapore; Singapore Immunology Network Singapore Singapore

The following is the authors’ response to the original reviews.

**Public Reviews:**

**Reviewer #1 (Public review):**

The manuscript presents a compelling new in vitro system based on isogenic co-cultures of human iPSC-derived hepatocytes and macrophages, enabling the modelling of hepatic immune responses with unprecedented physiological relevance. The authors show that co-culture leads to enhanced maturation of hepatocytes and tissue-resident macrophage identity, which cannot be achieved through conditioned media alone. Using this system, they functionally validate immune-driven hepatotoxic responses to a panel of drugs and compare the system's predictive power to that of monocyte-derived macrophages. The results underscore the necessity of macrophage-hepatocyte crosstalk for accurate modelling of liver inflammation and drug toxicity in vitro.

The manuscript is clearly written and addresses a key limitation in liver organoid systems: the lack of immune complexity and tissue-specific macrophage imprinting. Nevertheless, several conclusions would benefit from a more careful interpretation of the data, and some important controls or explanations are missing, particularly in the flow cytometry gating strategies, stress marker validation, and cluster interpretations.

Strengths:

(1) Novelty and Relevance: The study presents a highly innovative co-culture system based on isogenic human iPSCs, addressing an unmet need in modelling immune-mediated hepatotoxicity.

(2) Mechanistic Insight: The reciprocal reprogramming between iHeps and iMacs, including induction of KC-specific pathways and hepatocyte maturation markers, is convincingly demonstrated.

(3) Functional Readouts: The application of the model to detect IL-6 responses to hepatotoxic compounds enhances its translational relevance.

Weaknesses:

(1) Several key claims, particularly those derived from PCA plots and DEG analyses, are overinterpreted and require more conservative language or further validation.

We agree that PCA does not allow for maturation trajectories and mentioned that it was a hypothesis that the co-culture was promoting maturation, which we later validated by looking at the expression of key hepatocyte markers as well as by pearson correlation comparison with fetal hepatocytes.

(2) The purity of sorted hepatocytes and macrophages is not convincingly demonstrated; contamination across gates may confound transcriptomic readouts.

We agree and have highlighted and addressed this limitation in our discussion. Unfortunately, this is a limitation of bulk sequencing that a small amount of contamination might be present, however the TPM values of ALB for example in the iMacs is extremely low especially when compared to the hepatocytes, indicating that the level of contamination is likely to be very low. Likewise, the expression of CSF1R in the co-cultured iHeps is also extremely low. This has been included in Supp Fig 1F and G.

(3) Stress response genes and ER stress/apoptosis signatures are not properly assessed, despite being potentially activated in the system.

This has been included in Supp Fig 2C, where we’ve included the expression of ATF4, CASP3 and CASP9. Although there’s a significant difference in ATF4 expression between Day 0 and Day 7 iHep only/Co-culture, there is no significant difference between the Day 7 iHep only and Day 7 iHep Co-culture. There are no significant differences in CASP3 and CASP9 expression across all the samples.

(4) Some figure panels and legends lack statistical annotations, and microscopy validation of morphological changes is missing.

Although we agree that the morphology changes would be interesting, we think that this question is unfortunately outside of the scope of our question. Although Kupffer cells are in direct contact with hepatocytes, they migrate from the liver parenchyma into the sinusoidal spaces where they primarily reside. We do not think that the morphology would add much to the paper, especially given that this is a 2D model as well.

(5) The co-culture model with monocyte-derived macrophages is not fully characterised, making comparisons less informative.

Although we agree that it would be interesting to look more closely at the monocyte-derived macrophage co-cultures as well, we think that this would be more suited to a future study as the transcriptomic analysis would likely include confounding effects of patient specific transcriptomic changes, and our primary focus was on developing an isogenic co-culture system.

**Reviewer #2 (Public review):**

Summary:

This study builds on work by Glass and Guilliams showing that mouse Kupffer cells depend on the surrounding cells, including endothelium, hepatocytes, and stellate cells, for their identity. Herein, the authors extend the work to human systems. It nicely highlights why taking monocyte-derived macrophages and pretending they are Kupffer cells is simply misleading.

Strengths:

Many, including human cells, difficult culture assays, and important new data.

Weaknesses:

This reviewer identified minor queries only, rather than 'weaknesses' as such.

**Reviewer #3 (Public review):**

Summary:

In this study, the authors establish a human in vitro liver model by co-culturing induced hepatocyte-like cells (iHEPs) with induced macrophages (iMACs). Through flow cytometry-based sorting of cell populations at days 3 and 7 of co-culture, followed by bulk RNA sequencing, they demonstrate that bidirectional interactions between these two cell types drive functional maturation. Specifically, the presence of iMACs accelerates the hepatic maturation program of iHEPs, while contact-dependent cues from iHEPs enhance the acquisition of Kupffer cell identity in iMACs, indicating that direct cell-cell interactions are critical for establishing tissue-resident macrophage characteristics.

Functionally, the authors show that iMAC-derived Kupffer-like cells respond to pathological stimuli by producing interleukin-6 (IL-6), a hallmark cytokine of hepatic immune activation. When exposed to a panel of clinically relevant hepatotoxic drugs, the co-culture system exhibited concentration-dependent modulation of IL-6 secretion consistent with reported drug-induced liver injury (DILI) phenotypes. Notably, this response was absent when hepatocytes were co-cultured with monocyte-derived macrophages from peripheral blood, underscoring the liver-specific phenotype and functional relevance of the iMAC-derived Kupffer-like cells. Collectively, the study proposes this co-culture platform as a more physiologically relevant model for interrogating macrophage-hepatocyte crosstalk and assessing immune-mediated hepatotoxicity in vitro.

Strengths:

A major strength of this study lies in its systematic dissection of cell-cell interactions within the co-culture system. By isolating each cell type following co-culture and performing comprehensive transcriptomic analyses, the authors provide direct evidence of bidirectional crosstalk between iMACs and iHEPs. The comparison with single-culture controls is particularly valuable, as it clearly demonstrates how co-culture enhances functional maturation and lineage-specific gene expression in both cell types. This approach allows for a more mechanistic understanding of how hepatocyte-macrophage interactions contribute to the acquisition of tissue-specific phenotypes.

Weaknesses:

(1) Overreliance on bulk RNA-seq data:

The primary evidence supporting cell maturation is derived from bulk RNA sequencing, which has inherent limitations in resolving heterogeneous cellular states and functional maturation. The conclusions regarding hepatocyte maturation are based largely on increased expression of a subset of CYP genes and decreased AFP levels - markers that, while suggestive, are insufficient on their own to substantiate functional maturation. Additional phenotypic or functional assays (e.g., metabolic activity, protein-level validation) would significantly strengthen these claims.

We have added a discussion on the limitations of our study.

(2) Insufficient characterization of input cell populations:

The manuscript lacks adequate validation of the cellular identities prior to co-culture. Although the authors reference previously published protocols for generating iHEPs and iMACs, it remains unclear whether the cells used in this study faithfully retain expected lineage characteristics. For example, hepatocyte preparations should be characterized by flow cytometry for ALB and AFP expression, while iMACs should be assessed for canonical macrophage markers such as CD45, CD11b, and CD14 before co-culture. Without these baseline data, it is difficult to interpret the magnitude or significance of any co-culture-induced changes.

We apologise for this oversight, some of the markers were used in determining the purity of the iMacs before co-culture, and we did not end up including these plots for brevity. We have added the purity plots in Supp Fig 2E now, showing that the iMacs were more than 90% pure before co-culture. We acknowledge the concern about cross-contamination for bulk sequencing, and have added in Supp Fig 2G and H the expression of ALB in the iMac fraction, as well as the expression of CSF1R in the iHep fraction, showing minimal contamination with our gating strategy.

(3) Quantitative assessment of IL-6 production is insufficient:

The analysis of drug-induced IL-6 responses is based primarily on relative changes compared to control conditions. However, percentage changes alone are inadequate to capture the biological relevance of these responses. Absolute cytokine production levels - particularly in response to LPS stimulation - should be reported and directly compared to PBMC-derived macrophages to determine whether iMAC-derived Kupffer-like cells exhibit enhanced cytokine output. Moreover, the Methods section should clearly describe how ELISA results were normalized or corrected to account for potential differences in cell number, viability, or culture conditions.

We apologise if this was unclear. The cytokine production from dosed cells was normalized based on the viability of cells measured from the same well.

(4) Unclear mechanistic interpretation of IL-6 modulation:

The observed changes in IL-6 production upon drug treatment cannot be interpreted solely as evidence of Kupffer cell-specific functionality. For instance, IL-6 suppression by NSAIDs such as diclofenac is well known to result from altered prostaglandin synthesis due to COX inhibition, while leflunomide's effects are linked to metabolite-induced modulation of immune cell proliferation and broader cytokine networks. These mechanisms are distinct from Kupffer cell identity and may not directly reflect liver-specific macrophage function. Consequently, changes in IL-6 secretion alone - particularly without additional mechanistic evidence or analysis of other cytokines - are insufficient to conclude that co-culture with hepatocytes drives the acquisition of bona fide Kupffer cell maturity.

We fully agree with the reviewer and have highlighted this in our discussion.

**Recommendations for the authors:**

**Reviewer #1 (Recommendations for the authors):**

(1) GSE ID for RNA-seq data has not been provided.

This has been included.

(2) Line 291: Can the authors specify what they mean by "state-of-the-art"?

What we mean here is what others in the field have also recently described. We have rewritten this to be clearer.

(3) Lines 299-300: check sentence for grammar mistakes.

We have rewritten and clarified this.

(4) Figure 1B: The PCA does not really allow for following maturation trajectories. Also, all samples (day 3 Co-iHep, day 7 Co-iHep, day 7 iHep) look as if they cluster more or less together. Therefore, the conclusion drawn in lines 303-305 does not hold. Why is day 3 iHep not also shown here?

We agree that PCA does not allow for maturation trajectories and mentioned that it was a hypothesis that the co-culture was promoting maturation, which we later validated by looking at the expression of key hepatocyte markers as well as by pearson correlation comparison with fetal hepatocytes.

(5) Can the authors show that the cells that they are sorting in the double negative gate are indeed hepatocytes? Typically, these cells are big in cell size; therefore, showing the FSC/SSC gate would also be important.

We have added the FSC/SSC gate in supp fig. 1E to show that the populations have different sizes.

(6) Can the authors provide microscopy pictures of iHeps, iMacs, and the co-cultured cells for the reader to appreciate whether the morphology of cells already changes during the co-culture experiments?

Although we agree that the morphology changes would be interesting, we think that this question is unfortunately outside of the scope of our question. Although Kupffer cells are in direct contact with hepatocytes, they migrate from the liver parenchyma into the sinusoidal spaces where they primarily reside. We do not think that the morphology would add much to the paper, especially given that this is a 2D model as well.

(7) Please show expression of apoptotic and ER stress genes comparing Day7 iHeps and Co-iHeps, since genes such as c-Fos and Ppp2r3b can also be associated with cellular stress.

This has been included in Supp Fig 2C, where we’ve included the expression of ATF4, CASP3 and CASP9. Although there’s a significant difference in ATF4 expression between Day 0 and Day 7 iHep only/Co-culture, there is no significant difference between the Day 7 iHep only and Day 7 iHep Co-culture. There are no significant differences in CASP3 and CASP9 expression across all the samples.

(8) In addition to the genes shown in Figure 1E, could the authors extract a longer gene list of maturing hepatocytes and display them all in bar graphs or heatmaps, or similar? E.g., Albumin expression is shown later, but why not show it already here?

There are not many differences in the canonical hepatocyte markers, which is why we chose only to show the interesting genes that were different, as seen in the later ALB expression plot where there wasn’t a difference in ALB expression after 7 days of co-culture. Instead, we have included a new heatmap in Supp Fig 2B showing the top 40 genes that are contributing to the similarity by pearson correlation.

(9) Along these lines, how do the authors ensure that they are culturing only hepatocytes and do not have a mixture of cells that may "dilute" the hepatocyte signature?

Unfortunately, this is an limitation of our methodology, although the expression of key hepatic markers are routinely confirmed by qPCR to ensure that the majority of the cells are hepatocyte-like.

(10) Lines 347-350: similar to the interpretation of the PCA for hepatocytes, this is a completely random interpretation. The expression of ALB in the co-cultured iMacs indicates that there are some hepatocytes that ended up in the macrophage gate.

We agree and have highlighted and addressed this limitation in our discussion. Unfortunately, this is a limitation of bulk sequencing that a small amount of contamination might be present, however the TPM values of ALB for example in the iMacs is extremely low especially when compared to the hepatocytes, indicating that the level of contamination is likely to be very low. Likewise, the expression of CSF1R in the co-cultured iHeps is also extremely low. This has been included in Supp Fig 1F and G.

(11) Figure 2D: Among the pathways shown, there are also stress pathways (acute phase response, HMGB1). Also for these cells, control of apoptotic and ER stress signatures is necessary.

As mentioned, we have included some stress genes in Supp Fig 2C to address this.

(12) Lines 385-386: Why would FCGRA3 indicate tissue residency? Is there literature to support this statement?

CD16 is a marker often used to distinguish Kupffer cells from the surrounding cells, although it also expressed by non-classical monocytes, we have clarified the text here (Lines 356-357).

(13) Figure 3E: ALB and other genes were at the same or even lower levels expressed in D7 compared to D3. Why is that? Are the cells starting to de-differentiate after 7 days? Please discuss.

This is a very interesting question that we were wondering ourselves as well, although sadly we do not have an answer yet. We hypothesized that this might be due to the activation of cell proliferation/developmental programmes as the cells are kept longer together, as shown by the expression of morphogens like OSM and IGF-2 after co-culture. We have added some discussion for this (Lines 532-540)

(14) Line 459: Word "in" is double

We thank the reviewer for catching this, this has been corrected

(15) Figure 5: The findings are interesting, but the co-culture model remains somewhat unclear. Can the authors show, e.g., using qRT-PCR, how hepatocytes are developing in this culture system? If the development with monocyte-derived macrophages is altered, then one would expect that also the cellular response is different.

We agree with the reviewer, but we think that this question would be better answered in a follow-up study. We were looking to answer if the addition of isogenic iMacs would change the drug response of iHeps, and were using the PBMC-derived macrophages here as a control. A more complete study taking into account the genetic background of the donor PBMC-derived macrophages would be much more informative, but sadly outside of the scope of our present study.

(16) Lines 482-484: The authors talk about LPS-treated cultures and refer to Figure 4. However, there is no graph shown for LPS.

We apologise for being unclear here, but the co-cultures were co-treated with LPS during the drug stimulation assays, as it had been shown that LPS increases the sensitivity of the liver toward hepatotoxic drugs. We have clarified this in the main text (Lines 435-437).

**Reviewer #2 (Recommendations for the authors):**

(1) It would be nice to add some protein production by the hepatocytes. For example, can they produce albumin or some other protein that can be measured? Perhaps I missed this.

The protein expression of Albumin and Urea were assessed in the hepatocytes prior to co-culture in Supp Fig 1C; however we did not measure the protein level changes after co-culture as the co-culture would have a significant number of macrophages as well which we thought might affect the readout. Instead, after co-culture the primary analysis was done on the RNA levels of ALB and other cytochrome genes after sorting in Fig 3.

(2) Was there an increase in hepatocyte number? Did one cell outgrow the other, or did they maintain numbers?

The relative proportion of the iHeps remained the same, although we did see an expansion in the iMac population after 7 days by flow cytometry in Fig 1D.

(3) What happens if the iMACs and the iHeps are grown in Costar chambers with pore sizes too small to allow for cell contact, but allowing supernatant to be continuously exposed to both cell types?

We were primarily focused on the acquisition of KC-like phenotype in the iMacs with regards the question of direct contact, which was why we chose to use conditioned iHep media as part of the iMac experimental set up. However, it would be very interesting to see if the converse is also true, and whether secreted factors from the iMacs alone would be sufficient to drive the changes we observed in the iHeps after co-culture in a follow-up study.

(4) The discussion could use a brief paragraph on some limitations and what could be added to the co-culture system. For example, could stellate cells and sinusoidal endothelium also impart KC identity? Would growing KCs on endothelium provide a more natural substratum?

Once again, these are very interesting questions which are unfortunately outside of the scope of our study. However, we have included a short section discussing this in the paper, as we do think that it would be interesting to look at iMacs educated by hepatocyte vs stellate cells for example (Lines 530-536).

(5) The axonal guidance pathway in early iMACs is interesting. A recent report in vivo showed that macrophages migrate from the liver parenchyma into the sinusoids in neonates when they are still immature. The process could be chemotaxis, or it could be repulsion by parenchyma. Numerous axonal guidance molecules are repulsive, pushing axons away (robo/slit, etc). The migration of Kupffer cells into sinusoids could be a repulsive rather than a chemoattractant pathway. Did the RNA seq data provide any interesting molecules in this regard?

**Reviewer #3 (Recommendations for the authors):**

This manuscript presents a conceptually well-designed approach to modeling hepatocyte-macrophage crosstalk in vitro. The authors develop a co-culture system aimed at recapitulating key aspects of Kupffer cell (KC) identity and hepatocyte maturation. The data convincingly show that macrophages acquire KC-like features under co-culture conditions. However, several major issues limit the strength of the conclusions, the depth of mechanistic insight, and the translational impact of the work.

First, the study relies heavily on bulk RNA-seq data with minimal functional or protein-level validation - particularly for hepatocyte maturation. To substantiate claims of functional maturation, additional assays measuring albumin secretion, urea production, and CYP activity are essential. Furthermore, the omission of zonation-associated markers (e.g., GLUL, CPS1, CYP2E1) leaves a critical gap in assessing whether the iHEPs achieve physiologically relevant functional states.

Second, statistical interpretation and reporting are inconsistent. Significant and non-significant findings are frequently conflated, which risks overinterpretation. For instance, the reported reduction in HNF4A expression is not statistically significant, and AFP expression is only significantly reduced in Day 7 co-iHEPs - yet these distinctions are not clearly stated.

Third, although the authors emphasize the role of cell-cell contact in promoting KC identity, no experiments (e.g., transwell separation, adhesion-blocking assays) directly test this claim. As a result, the mechanistic basis for this conclusion remains speculative.

Finally, while the data support enhanced macrophage differentiation toward a KC-like phenotype, the evidence that co-culture significantly promotes hepatocyte maturation is far less convincing and requires additional functional, mechanistic, and statistical validation before firm conclusions can be drawn.

Minor comments:

(1) Methodology: The choice of a 2.5:1 iHEP:iMAC ratio is not justified. This proportion does not reflect physiological hepatocyte-to-KC ratios in vivo and should be either rationalized or benchmarked against native liver composition.

We admit that the ratio here is on the higher side of things, but it has been previously reported that there can be between 20 to 40 macrophages per 100 hepatocytes (1:5 to 1:2.5) in the adult mouse liver (Baratta et al., 2009), while admittedly in the developing mouse liver the ratio is closer to 1:4 (Lopez et al., 2011). We chose 1:2.5 as we anticipated that not all of the macrophages would be able to attach, and would thus be lost during media change, as evident by the flow cytometry of the co-culture on Day 3 of the co-culture, where only 20% of the cells had clear CD45 and CD14 expression. We have clarified our methodology in paper (Lines 141-143).

(2) Effect of iMAC on iHEP (Section 3.2, Supplementary Figure 1E):

(2.1) The authors should explain why Day 3 co-cultured iHEPs show stronger transcriptomic similarity to primary hepatocytes than Day 7 cells. Possible biological mechanisms (e.g., transient paracrine signaling or temporal changes in maturation dynamics) should be discussed.

We have added some discussion for this (Lines 309-311, 536-540).

(2.2) The figure legend refers to "fetal hepatocytes," while the correlation map states "hepatocytes." This discrepancy must be clarified. Moreover, if fetal hepatocytes are used as the reference, and the goal is to assess maturation, comparisons to adult hepatocytes are necessary.

The comparison was done against fetal hepatocytes, and has been clarified in the figure. We chose to use fetal hepatocytes here as it would be unfair to compare iPSC-derived cells that are less than 3 weeks old to adult human tissue, and any similarity or differences between the mono/co-cultures to the adult tissue might be due to the shifting transcriptomic landscape during development. However, we do recognise the nuanced nature of using “maturation” here, and what we mean is that the iPSC-derived cells become more similar to their *in-vivo* counterparts.

(2.3) Baseline characterization of both cell types before co-culture is insufficient. For iHEPs, flow cytometry data on ALB and AFP positivity rates should be presented, along with post-co-culture changes. For iMACs, marker expression (CD45, CD11b, CD14) should be shown before and after co-culture. The methods mention CD163, CX3CR1, and CD11b, but these data are absent from the results. Additionally, the gating strategy for cell sorting prior to bulk RNA-seq must be clearly described - including how potential cross-contamination of cell fractions (e.g., macrophages in the hepatocyte population) was excluded.

We apologise for this oversight, some of the markers were used in determining the purity of the iMacs before co-culture, and we did not end up including these plots for brevity. We have added the purity plots in Supp Fig 2E now, showing that the iMacs were more than 90% pure before co-culture. We acknowledge the concern about cross-contamination for bulk sequencing, and have added in Supp Fig 2G and H the expression of ALB in the iMac fraction, as well as the expression of CSF1R in the iHep fraction, showing minimal contamination with our gating strategy.

(3) IGF2 Expression: The observed upregulation of IGF2, a fetal marker, contradicts the conclusion that co-culture promotes hepatocyte maturation. This inconsistency should be addressed, and possible explanations (e.g., transient fetal-like activation driven by macrophage-derived signals) discussed. The lack of statistical significance for this finding must also be explicitly noted.

We thank the reviewer for pointing this out. The expression of IGF2 was actually significantly different when comparing the Day 0 Hepatocyte only and Day 7 Hepatocyte only to the Day 3 Co-cultured Hepatocytes, but the significance is lost with the Day 7 co-cultured Hepatocytes. One possible explanation is as the reviewer suggested, that there is a transient program that is activated upon co-culture that is subsequently downregulated. We have updated the figure and text, and added some discussion to reflect this (Lines 309-311, 536-540).

(4) Effect of iHEP on iMAC: The reported upregulation of KC-related genes is overstated. Changes in LYVE1 and ID1 are not statistically significant (Figure 2G), yet they are presented as meaningful. Clear separation of statistically significant results from non-significant trends is critical to avoid overinterpretation.

We apologise for this, as it was never our intention to present these markers as significant, but rather we presented these markers because we thought that these markers would be of interest to the audience. We have clarified the text to reflect that these are trends and non-significant (Lines 367-369).

(5) Mimicking In Vivo Clinical Responses:

(5.1) The authors' conclusion that IL-6 responses are not recapitulated when iMACs are replaced by monocyte-derived macrophages (MoMs) is not fully supported by the data presented. In fact, the MoM co-cultures exhibit a noticeable trend toward increased IL-6 production (e.g., approximately 150% with LTG at 66.6 µM and 400 µM), suggesting that some degree of responsiveness is retained. To substantiate the claim that the observed cytokine modulation is unique to iKC-containing co-cultures, the authors should perform direct statistical comparisons of absolute IL-6 secretion levels between iKC and MoM co-cultures at each drug concentration. Such analyses are essential to determine whether the differences are statistically significant and biologically meaningful, and to clarify whether the observed effects truly reflect KC-specific functionality rather than general macrophage activation.

(5.2) The effects of drug exposure on hepatocytes themselves are not addressed. It is important to evaluate whether the co-culture remains viable under treatment, whether it recovers after drug withdrawal, and whether there is evidence of cytotoxicity or irreversible phenotypic loss.

(6) Interpretation of IL-6 Modulation and Model Specificity:

The authors show that IL-6 secretion in their co-culture system varies in response to multiple hepatotoxic drugs and parallels some reported clinical trends - notably, a concentration-dependent decrease with diclofenac (DIC) and leflunomide (LFM). They further report that this pattern is not observed in hepatocyte-PBMC-derived macrophage co-cultures, and they conclude that iMAC/iKC-like cells are essential for capturing immune-mediated hepatotoxic responses. However, the data presented do not fully justify such a conclusion. Several key mechanistic issues weaken the interpretation:

(6.1) Mechanistic ambiguity in the DIC response: The decrease in IL-6 following DIC exposure is most likely attributable to reduced prostaglandin E₂ (PGE₂) production via COX inhibition, which secondarily suppresses IL-6 signaling. This effect is a general pharmacological property of NSAIDs and is not necessarily reflective of Kupffer cell-specific pathways. Direct evidence - such as prostanoid quantification or PGE₂ rescue experiments - is required to establish that the observed effects are liver-specific rather than nonspecific NSAID responses.

(6.2) Pharmacogenetic complexity in the LFM response: LFM-induced hepatotoxicity is highly variable and largely dependent on CYP2C9 polymorphisms, which determine conversion to the active metabolite teriflunomide. Because hepatotoxicity and the associated cytokine responses are not universal among patients, a simplified co-culture model lacking metabolic diversity cannot be assumed to faithfully reproduce patient-specific immune responses. The observed IL-6 suppression could arise from differences in metabolic activation, intracellular exposure, or indirect signaling changes rather than from intrinsic KC-specific mechanisms.

These points significantly undermine the authors' claim that IL-6 modulation provides definitive evidence of model specificity or predictive value. At minimum, the manuscript should (i) explicitly acknowledge these mechanistic limitations, (ii) include supporting data such as prostanoid profiling, CYP2C9 modulation, or teriflunomide quantification, and (iii) temper its claims regarding the model's capacity to recapitulate immune-mediated hepatotoxicity. Without such evidence, the current interpretation risks overstating the functional significance and translational relevance of the co-culture system.

We fully agree with the reviewer and have highlighted this in our discussion (Lines 540 – 551).
